# PRX102 Participates in Root Hairs Tip Growth of Rice

**DOI:** 10.1186/s12284-023-00668-7

**Published:** 2023-11-16

**Authors:** Sunok Moon, Behnam Derakhshani, Yun Shil Gho, Eui-Jung Kim, Su Kyoung Lee, Xu Jiang, Choonseok Lee, Ki-Hong Jung

**Affiliations:** Graduate School of Green-Bio Science and Crop Biotech Institute, Kyung Hee University, Yongin, 17104 Korea

**Keywords:** Endoplasmic reticulum, Peroxidase, Polar tip growth, RNA-seq analysis, Root hair

## Abstract

Root hairs are extensions of epidermal cells on the root tips that increase the root contract surface area with the soil. For polar tip growth, newly synthesized proteins and other materials must be incorporated into the tips of root hairs. Here, we report the characterization of PRX102, a root hair preferential endoplasmic reticulum peroxidase. During root hair growth, PRX102 has a polar localization pattern within the tip regions of root hairs but it loses this polarity after growth termination. Moreover, PRX102 participates in root hair outgrowth by regulating dense cytoplasmic streaming toward the tip. This role is distinct from those of other peroxidases playing roles in the root hairs and regulating reactive oxygen species homeostasis. RNA-seq analysis using *prx102* root hairs revealed that 87 genes including *glutathione S-transferase* were downregulated. Our results therefore suggest a new function of peroxidase as a player in the delivery of substances to the tips of growing root hairs.

**Supplementary Information:**

The online version contains supplementary material available at 10.1186/s12284-023-00668-7.

## Background

Root hairs are extensions of specific root epidermal cells that engage in tip growth. Root hairs play pivotal roles in nutrients and water uptake from the rhizosphere, and function as the site of interactions between roots and soil microorganisms (Dazzo et al. 1984; Gilroy and Jones 2000; Leavitt 1904). Root hair development is a genetically controlled process but is also flexibly modulated by environmental conditions (Bates and Lynch 2000; Datta et al. 2011; Kwasniewski et al. 2013; Ma et al. 2001; Muller and Schmidt 2004). Plants fine-tune root hair development to facilitate adaption to various environments, including low phosphate and potassium availability (Giri et al. 2018; Kumar et al. 2020; Verma et al. 2018; Yang et al. 2020a).

In *Arabidopsis*, epidermal cell fates are determined by cell position. A root hair arises from epidermal cell above the junction of two cortical cells, while non-hair cell is located over a single cortex cell, creating the separated longitudinal files of root hairs and non-hair cells (Marzec et al. 2014). MYB-bHLH-WD40 complex is involved in the fate decisions in *Arabidopsis* root epidermis (Wei and Li 2018). Root hairs are shorter than non-hair cells throughout their development, from the division zone to the maturation zone (Scheres et al. 2002). While in rice, any cells on the root epidermis can produce root hairs, but only some develop into root hair cells (Salazar-Henao et al. 2016). Furthermore, the factors determining root epidermal cell fate remain unknown. Root hairs are shorter than non-hair cells at the maturation zone, which is due to by slow expansion of trichoblasts after hairs initiation (Marzec et al. 2014; Scheres et al. 2002). Unlike fate determination, root hair tip growth is well conserved between *Arabidopsis* and rice. RHD6-LIKE (RSL) transcription factors, which are key regulators of root hair growth, control reactive oxygen species (ROS) production and promote root hair elongation in both *Arabidopsis* and rice (Mangano et al. 2017; Moon et al. 2019; Kim et al. 2017).

ROS also play essential roles during root hair development. *RHD2*, *RHT5* and *OsNOX3* encode NADPH oxidase in *Arabidopsis*, maize and rice, respectively, where they are involved in production of apoplastic superoxide ion (O^2−^) (Foreman et al. 2003; Monshausen et al. 2007; Nestler et al. 2014; Wang et al. 2018). In *rhd2*, *rht5*, and *osnox3* mutants, tip focused ROS accumulation was not detected and the length of root hairs was reduced, indicating that ROS drives root hair elongation (Foreman et al. 2003; Monshausen et al. 2007; Nestler et al. 2014; Wang et al. 2018). In cells, O^2−^ is rapidly converted into hydrogen peroxide (H_2_O_2_), either spontaneously or by superoxide dismutase (Zhao et al. 2016). However, apoplastic H_2_O_2_ can be generated by many enzymes, including oxalate oxidase, diamine oxidase, and Class III peroxidases (Caliskan and Cuming 1998; Federico and Angelini 1986; Elstner and Heupel 1976; Dunand et al. 2007). Apoplastic H_2_O_2_ plays another role as a second messenger; it can move into the cytoplasm via aquaporin or can be sensed by specific receptor like kinases (Wu et al. 2020; Bienert and Chaumont 2014). Finally, apoplastic H_2_O_2_ is detoxified via both antioxidants and enzymatic reactions (Podgorska et al. 2017; Wu et al. 2020; Bienert and Chaumont 2014).

Plants have two kind of peroxidases: Class I and Class III (Welinder 1992). Class I peroxidases are intracellular proteins while Class III peroxidases are secreted into the extracellular space or are transported into the vacuole (Yang et al. 2020b; Shigeoka et al. 2002). Class III peroxidases exist as a multi-gene family of enzymes that are involved in diverse plant functions, including defense, lignification, and auxin catabolism (Cosio et al. 2009). Class III peroxidases execute two opposite roles in the ROS pool, reducing H_2_O_2_ or producing hydroxyl radicals (OH^−^) (Marjamaa et al. 2009; Raggi et al. 2015; Kidwai et al. 2020). In root hairs, PRX01, PRX44, PRX73, PRX62, and PRX69 trigger root hair growth via regulation of ROS homeostasis and solubilization of cell wall extensins (Pacheco et al. 2022; Marzol et al. 2022).

In this paper, we identify a peroxidase gene, *PRX102*, that is preferentially expressed in root hairs, where it regulates root hair tip growth. PRX102 also participates in root hair outgrowth by regulating the dense cytoplasmic streaming toward the tip, and not by dramatic regulation of the ROS pool. In this study, we suggest a new function of peroxidase related to delivery of substances to the tip of the growing root hair.

## Results

### PRX102 Exhibited Root Hairs-Defective Phenotype

**Fig. 1:**
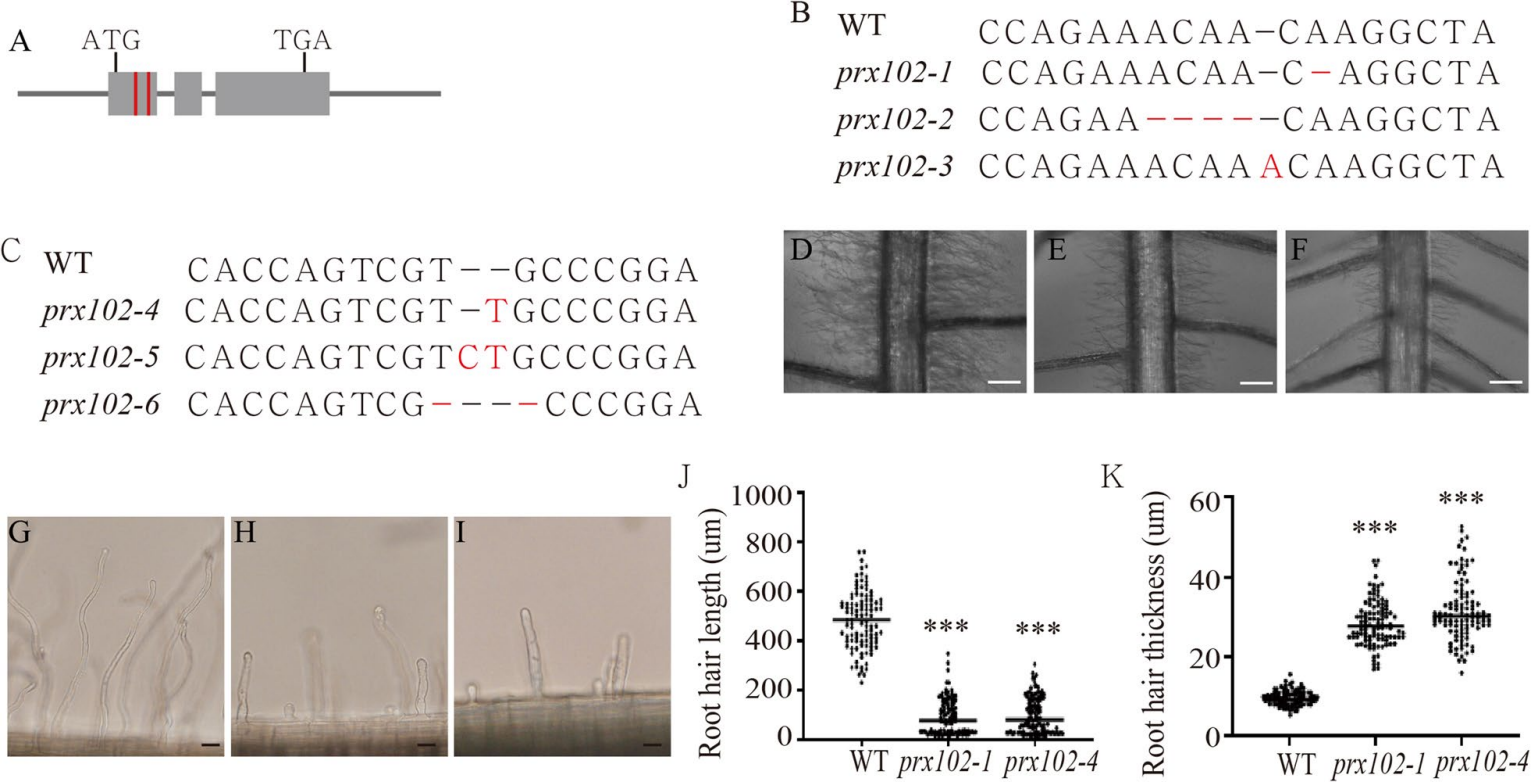
Phenotypic analysis of *prx102* using gene-editing mutants. Shown are: **A**, Schematic representation of the *PRX102* gene, including target regions for the construction of CRISPR/Cas9 vectors (Red bar). **B** and **C**, Mutated sequence analysis of target regions within the first exon of *PRX102*. Black letters indicate sequences of target regions in the WT, and red letters indicate missed or added sequences of target regions in *prx102*. Mutant phenotypes of *PRX102*: WT (**D** and **G**), *prx102-1* (**E** and **H**), and *prx102-4* (**F** and **I**). Also shown are: root hair length (**J**) and width (**K**) measured at 1 cm from the apex of WT and *prx102* plants (n =  > 100 root hairs). Scale bars in **D**–**F** = 1 mm; Scale bars in **G**–**I** = 30 µm

In a previous study, we performed a genome-wide analysis of root hair-preferential genes in rice (Moon et al. 2018). To identify the key genes responsible for root hair development, we generated mutant lines for four known root hair-preferential genes using the CRISPR/Cas9 system (Additional file 1: Table S1). Using sequencing analysis, we selected a homozygotic mutant for each gene and observed the morphology of root hairs five days after germination (DAG). Using this screening, we identified a root hair defective mutant possessing a mutation within *PEROXIDASE102* (*PRX102, LOC_Os07g31610*), a gene coding a protein comprised of three exons (Fig. 1A).

To check whether mutations within *PRX102* were responsible for the root hair defective phenotype, we analyzed *PRX102* sequences from T1 lines edited using a CRISPR/Cas9 gene editing protocol. We identified three homozygotic mutants with small deletions or one base insertions within *PRX102* and called them *prx102-1* to *prx102-3* (Fig. 1B). To confirm the mutant phenotype, additional alleles of *PRX102* were generated using another CRISPR/Cas9 construct; we obtained three monoallelic mutants (i.e., *prx102-4* to *prx102-6*) (Fig. 1A and C). Root hairs from *prx102* are shorter and thicker than those of the wild type (WT) (Fig. 1D–I). Next, for quantitative analysis we measured root hair lengths 1 cm from the apex. Root hair length was reduced by 80% and 79% in the *prx102-1* and *prx102-4* mutants relative to the WT. Moreover, root hair thickness was increased by 2.9 fold with respect to the WT in *prx102-1* and *prx102*-4 (Fig. 1J and K). Taken together, these data indicate that PRX102 plays an essential role in root hair tip growth in rice.

### PRX102 is Preferentially Expressed in Trichoblasts and Root Hairs

**Fig. 2:**
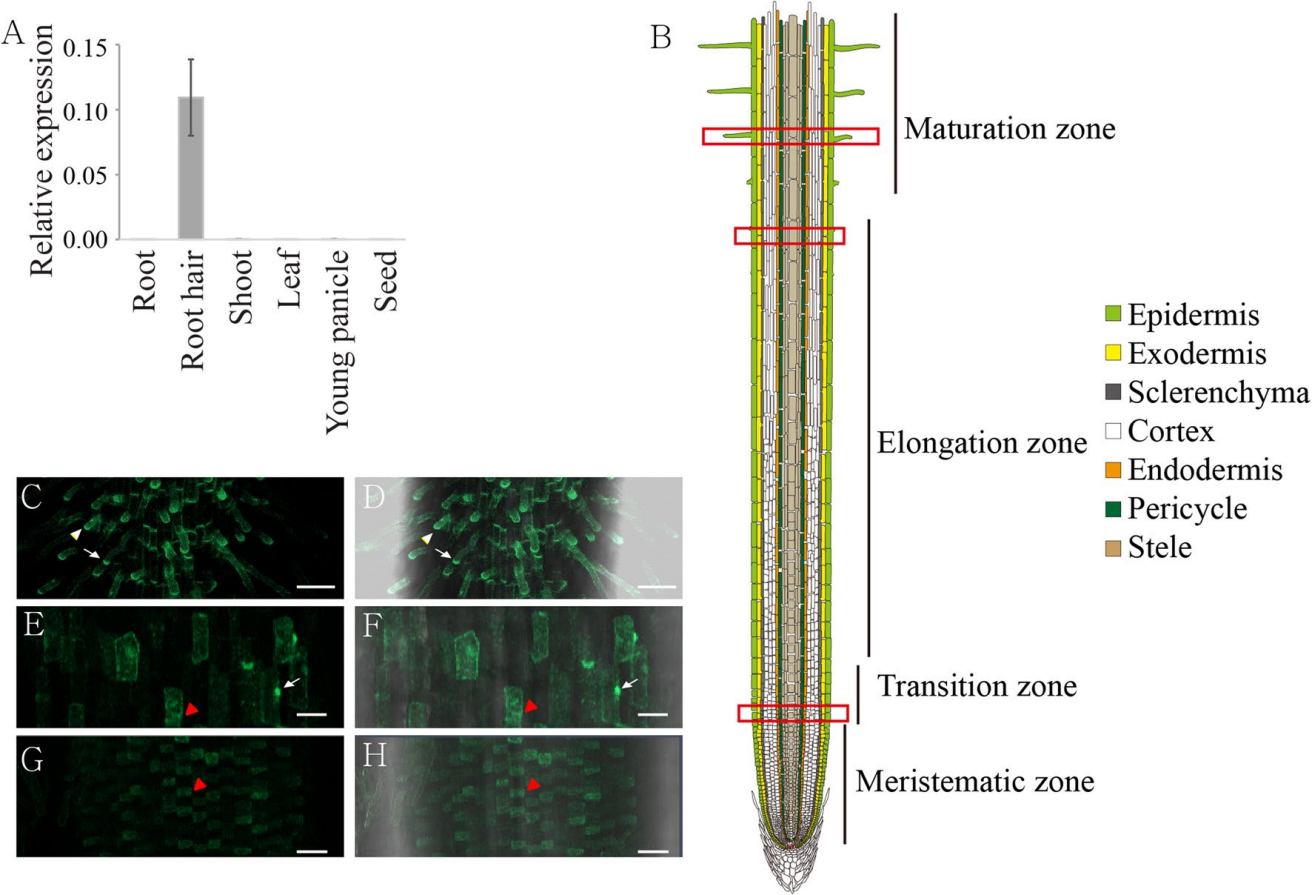
Expression pattern analysis of *PRX102*. Shown are: **A**, RT-qPCR analysis of *PRX102* in various tissues of rice plants. **B**, Longitudinal view of rice roots. Four distinct regions with different growth activities are represented as meristematic zone, transition zone, elongation zone, and maturation zone. Red boxes indicate the position of root shown in **C**–**H**. **C**–**H**, Fluorescent images from transgenic plants carrying the pPRX102::PRX102–GFP cassette. Photos were taken of the maturation zone (**C** and **D**), the elongation zone (**E** and **F**), and the transition zone of roots (**G** and **H**). GFP signals were detected in root hairs (white arrow heads, **C** and **D**) and trichoblasts (red arrow heads, **E**–**H**). PRX102–GFP accumulated at tips of growing root hairs (**C** and **D**) and bulge of trichoblasts (**E** and **F**) (white arrows). Fluorescent images (**C**, **E**, and **G**) and blight-field merged images (**D**, **F**, and **H**). Scale bars in **C** and **D** = 100 µm; scale bars in **E**–**H** = 20 µm

Next, we performed quantitative RT-PCR to validate the root hair-preferential expression pattern of *PRX102*. Whereas *PRX102* was abundantly expressed in root hairs, it was seldom detected in other organs (i.e., roots, shoots, mature leaves, young panicles, and developing seeds) (Fig. 2A). Along longitudinal axis of root, the root divided four regions: meristematic zone, transition zone, elongation zone, and maturation zone (Fig. 2B). The division zone consists of cells which divide as an undifferentiated form. After division in the meristematic zone, cells enter the transition zone where they undergo physiological changes to prepare for rapid elongation (Verbelen et al. 2006). Cells elongate rapidly in the elongation zone, but growth rates decrease to zero at the maturation zone (Fig. 2B) (Dolan and Davies 2004). To know exact expression pattern of *PRX102 in planta*, we generated transgenic plants harboring *PRX102* promoter::PRX102–GFP vector. GFP signals were strongly detected in root hairs of maturation zone and trichoblasts of elongation zone (Fig. 2C–F). PRX102-GFP especially accumulated at the tips of root hairs and bulging region of trichoblasts (Fig. 2C– F). GFP signals were first detected in trichoblasts at the transition zone of the root before epidermal elongation (Fig. 2G and H) (Lavrekha et al. 2017).

### PRX102 Exhibits Polarized Localization During Root Hair Growth

**Fig. 3:**
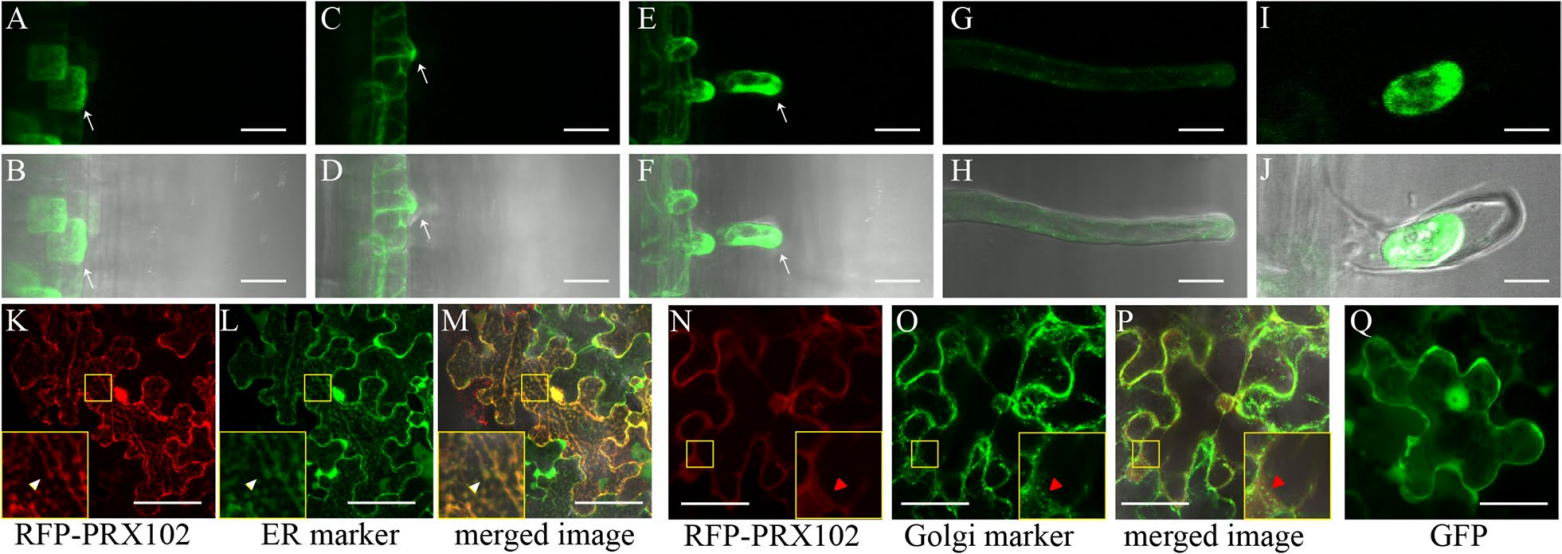
Localization analysis of PRX102. Shown are: **A**–**H**, Subcellular localization of PRX102 in transgenic plants carrying the pPRX102::PRX102–GFP cassette. Photos were taken at the transition zone (**A** and **B**), the elongation zone (**C** and **D**), and the maturation zone of the root (**E**–**H**). GFP images in root hairs at fast growing stage (**E** and **F**) and growth termination stage (**G** and **H**). Arrows indicate accumulated GFP signal at trichoblasts root hairs. **I** and **J**, Localization of PRX102 after plasmolysis using 500 mM mannitol. Fluorescent images (**A**, **C**, **E**, **G**, and **I**) and blight-field merged images (**B**, **D**, **F**, **H**, and **J**). RFP–PRX102 (**K** and **N**), ER marker (**L**), Golgi marker (**O**) and merged image (**M** and **P**) in leaf epidermal cells from *Nicotiana benthamiana*. Large yellow boxes at bottom left corner of **K**–**P** are the magnification of small yellow boxes in each image. White and red arrowheads mark overlapping signals and non-overlapping signals, respectively. Also shown are cells expressing 35S::GFP as control (**Q**). Scale bars = 20 μm (**A**–**H**), 10 μm (**I**–**J**), and 50 μm (**K**–**Q**)

In the transition zone, PRX102 is expressed in trichoblasts and is distributed in more abundance at the external region of root epidermal cells (Fig. 3A and B). After bulge formation in elongation zone, PRX102 becomes more polarly distributed toward the root hair initiation site (Fig. 3C and D). The GFP signal was mainly detected in root hair tips during root hair growth, but polarity was lost following termination of growth (Fig. 3E–H). Since most reported Class III peroxidases function in the apoplastic region or the cell walls of root hairs, we checked whether PRX102 is exported from cells. After plasmolysis using 500 mM mannitol, we observed that GFP signals remained within cells, indicating that PRX102 is not transported outside cells (Fig. 3I and J).

Next, to determine the exact location of PRX102 within cells, we conducted tobacco infiltration experiments. Moreover, since PRX102 appeared to be localized to the endoplasmic reticulum (ER) or trans-Golgi network in root cells, *Agrobacteria* harboring the RFP–PRX102 plasmid were co-transformed with *Agrobacteria* harboring GFP–HDEL (an ER marker), or mannosidase I–GFP (a Golgi marker), respectively (Berson et al. 2014; Sun et al. 2020). We observed that the RFP signal matched the ER marker (Fig. 3K–M). The enlarged image in the yellow box indicates that the signal of RFP-PRX102 completely overlaps that of the ER marker (Fig. 3K–M). However, when *Agrobacteria* harboring the RFP–PRX102 plasmid were co-transformed with *Agrobacteria* harboring with Golgi markers, the signals did not completely overlap (Fig. 3N–P). From images completely overlapping with ER markers, we infer that PRX102 is localized to the ER. On the other hand, we detected control GFP signals in both the cytosol and nucleus (Fig. 3Q).

### RNA-seq Analysis of *prx102* and WT Root Hairs Identifies Candidate Genes Involved in Root Hair Development

**Fig. 4:**
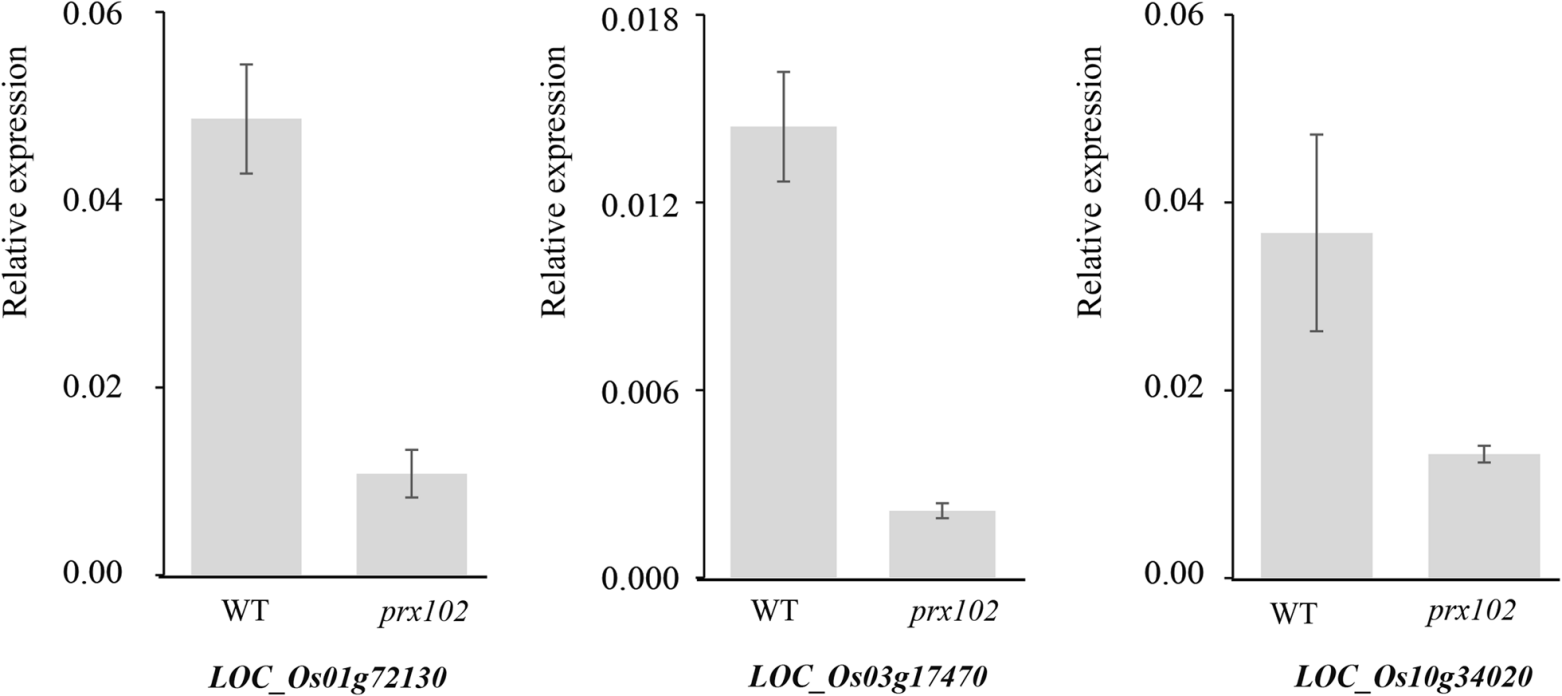
Real-time RT-qPCR analysis of three *glutathione S-transferase* genes found to be downregulated in the *prx102* mutant. Shown are the relative expression levels of *LOC_Os01g72130*, *LOC_Os03g17470,* and *LOC_Os10g34020* in WT and *prx102* root hairs. Error bars represent means ± SE (N = 3 replicates, root hairs sampled from more than 1000 seedlings)

To identify genes influenced by PRX102, we performed an RNA-seq analysis of root hairs from WT and *prx102*. In total, 87 genes were identified as downregulated genes using the following criteria: *P* value ≤ 0.05, FPKM value ≥ 4, and downregulation of at least 1.2 log2 (Additional file 1: Table S2). We then performed GO enrichment analysis of the biological process category using ShinyGO (http://bioinformatics.sdstate.edu/go/) (Ge et al. 2020). We found that the glutathione metabolic process GO term was significantly enriched among downregulated genes in *prx102*, exhibiting 18.6-fold enrichment (Table 1). Three *glutathione S-transferases* and one *glutathione synthase* were identified as involved in glutathione metabolic processes. We therefore confirmed the reduced expression of the three *glutathione S-transferases* via RT-qPCR analysis (Fig. 4). Consistent with the RNA-seq analysis results, three *glutathione S-transferase* genes (*LOC_Os01g72130*, *LOC_Os03g17470*, and *LOC_Os10g34020*) were down-regulated by more than two-fold in the root hairs from *prx102*, compared to wild type.

**Table 1:** Analysis of significantly enriched Gene Ontology terms of genes downregulated in *prx102* root hairs relative to the WT

GO name	Ref number^a^	Query number^b^	Fold enrichment^c^	Genes
Glutathione metabolic	96	4	18.6	LOC_Os01g72130 LOC_Os03g17470 LOC_Os10g34020 LOC_Os12g16200

Total number of GO terms in the rice genome is 35,671 and the number of GO terms associated with the genes downregulated in *prx102* is 85

^a^Selected GO Slim terms annotated in the rice genome

^b^The number of selected GO Slim terms in queried genes downregulated in *prx102*

^c^Relative ratio of observed to expected number of genes for a selected GO Slim term

### PRX102 is Not a Major Regulator of the ROS Pool in Root Hair

Peroxidases catalyze substrate oxidation by hydrogen peroxide or an organic peroxide (Passardi et al. 2004). Using a fluorescent hydrogen peroxide indicator (i.e., Peroxy Orange 1), we checked whether ROS levels were altered in the root hairs of *prx102* relative to the WT. We did not detect significant differences in signal intensity between the root hair of *prx102* and the WT (Additional file 2: Fig. S1) (Passardi et al. 2004; Gayomba and Muday 2020). This result indicates that PRX102 does not play a significant role in maintaining the ROS pool in root hair.

### Thin Cytoplasm Fluid is Observed in Tip of Root Hairs in *prx102*

The tip region of a growing root hair is filled with dense cytoplasm that contains many secretory vesicles originating from the ER. To determine why *prx102* root hairs were short, we stained roots using ER-Tracker™ Red. Unfortunately, ER staining caused ER aggregation in rice root hairs under our experimental conditions (Additional file 2: Fig. S2).

**Fig. 5:**
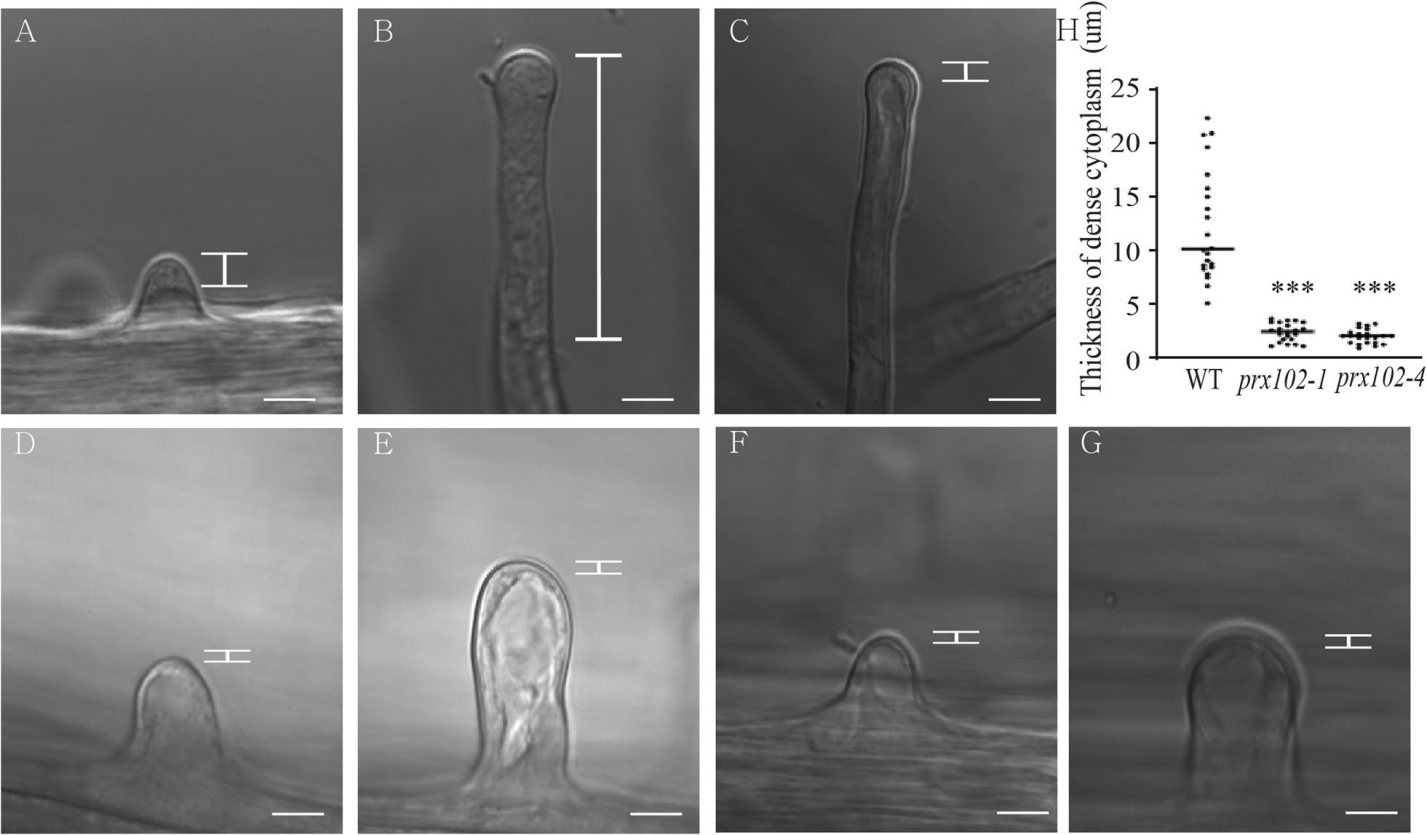
Dense cytoplasm in the tips of growing root hairs. **A**–**C**, Tip-concentrated dense cytoplasm in WT root hairs following initiation of polar growth (**A**), active growth stage (**B**), and termination of growth (**C**). Rare cytoplasm in the tips of root hairs from *prx102-1* (**D** and **E**) and *prx102-4* (**F** and **G**) after initiation of polar growth (**D** and **F**) and at the active growth stage (**E** and **G**). The white bar indicates regions of dense cytoplasm. Bars = 10 µm. H, The thickness of dense cytoplasm in tip of root hairs of WT and *prx102* plants. For measurement, a picture with thinnest cytoplasm was selected from ten photos taken at three-second intervals (n = 22 root hairs)

Next, we examined root hair morphologies carefully under a light microscope. In the WT, reverse fountain-like cytoplasm streaming was observed in root hair tips during polar growth, thereby exhibiting a tip-concentrated cytoplasmic distribution (Fig. 5A and B; Additional file 3: Video S1 and S2). At the mature stage, the density of the cytoplasm in the tip region was reduced (Fig. 5C; Additional file 3: Video S3). By monitoring GFP signals in transgenic plant having PRX102 promoter::PRX102–GFP, we found PRX102 moves along with cytoplasmic streaming (Video S4). We also observed cytoplasmic streaming in *prx102* (Video S5-S8). But a clear difference was found in the density of the cytoplasm between WT and *prx102.* In *prx102,* the layer of compact cytoplasm was thinner than in the WT at the tip growth stage, indicating that PRX102 is involved in the tip-concentrated cytoplasmic distribution in root hairs (Fig. 5D–G; Additional file 3: Video S5-S8). Because the thickness of the dense cytoplasm at the tip is continuously changed along to the cytoplasmic streaming, ten pictures were taken at three-second intervals for quantitative analysis. We used a picture with thinnest cytoplasm to measure thickness (Fig. 5H). Although the thicknesses of the cytoplasm at tip were not measured uniformly in the wild type owing to continuous movement of the cytoplasm, thickness of the dense cytoplasm was reduced in *prx102*.

## Discussion

### Glutathione Metabolic Processes are Downregulated in *prx102*

We observed that the *prx102* mutant exhibited short root hairs and a reduction in the dense cytoplasm of the root hair tip. We then performed and RNA-seq analysis to obtain more information regarding the role of PRX102 during root hair development. Functional analysis of the results of this RNA-seq analysis revealed that GO terms related to glutathione metabolic process were significantly enriched among genes that were downregulated in *prx102*. Among these genes, three *glutathione S-transferases* and one *glutathione synthase* were identified as involved in glutathione metabolic process. Glutathione is a tripeptide composed of cysteine, glutamic acid, and glycine. Although glutathione functions as a major antioxidant, it also plays other biological roles. Previous studies of *AtGSTF2* and *TgGST2* suggest that *glutathione S-transferase* plays a role in root hair development or vesicle transfer (Mang et al. 2004; Li et al. 2021). Moreover, ethylene is known to promote root hair growth and AtGSTF2 has been identified as an ethylene-induced *glutathione S-transferase* (Mang et al. 2004). Although the function of AtGSTF2 remains unknown, it is known to be expressed during root epidermis formation in *Arabidopsis* (Mang et al. 2004). *TgGST2* from *Toxoplasma gondii* is known to be involved in secretory vesicle trafficking and fusion, and a knockout mutant was found to show significantly reduced invasion capacity (Li et al. 2021).

### PRX102 Participates in Root Hair Tip Growth

PRX102 is a Class III peroxidase and its knockout mutant exhibits a short root hair phenotype. In total, the rice genome encodes at least 138 Class III peroxidase genes, of which more than thirteen are preferentially expressed in root hairs (Moon et al. 2018). Furthermore, several Class III peroxidases participate in tip growth in root hairs, where they regulate ROS homeostasis and the solubilization of cell wall extensins (Pacheco et al. 2022; Marzol et al. 2022).

During tip growth, newly synthesized proteins and other material are transported and incorporated into the tips of root hairs (Moon et al. 2022; Weng et al. 2023; Lombardo and Lamattina 2012). ER contributes driving force for cytoplasmic streaming which assists in delivering materials to growing tip (Liu et al. 2017). ER is concentrated in the dense cytoplasm of the subapical region of growing root hairs (Sieberer et al. 2002; Sun et al. 2020). A reduction in the dense cytoplasmic area at the tips of root hairs in *prx102* may therefore be due to a malfunction of the ER. In *prx102*, dense cytoplasm is restricted, and this may cause scarcity of material at the tips of the root hairs, which would eventually inhibit root hair growth.

RHD3, an ER-localized dynamin-like Atlastin GTPase, is known to be important in ER organization during root hair tip growth (Chen et al. 2011; Sun et al. 2020). Using an *rhd3* associated GFP marker, Qi et al. (2016) revealed that alteration of the ER structure causes a defect in root hair growth. In this study, we were able to explain the function of PRX102 by monitoring organelles in the root hairs of *prx102*. However, staining of the ER did not reveal the native ER structure due to aggregation. To further characterize the function of PRX102, future studies should express organellar fluorescence markers in the *prx102* background. Taken together, the data from this study suggests that peroxidase may play a new function related to the delivery of substances to the tips of growing root hairs.

## Methods

### Vector Construction and Rice Transformation

Mutants for four root hair-preferential genes were generated using CRISPR/Cas9 gene editing. To do so, 20-bp target sites were selected using CRISPRdirect and were then inserted into a pRGEB32 vector (AddGene plasmid ID: 63,142) (Naito et al. 2015). The primer sequences used for the construction of CRISPR/Cas9 vectors are listed in Additional file 1: Table S3. Next, using *Agrobacterium*-mediated transformation, we obtained transgenic plants. DNA was then extracted from transgenic plants and PCR was performed using gene specific primers (Additional file 1: Table S3). To verify the mutations were present, the PCR products were then sequenced (Macrogen, Seoul, Korea).

The promoter region and full-length cDNAs of PRX102 were amplified by PCR and assembled into the binary vector P1(Additional file 1: Table S3). Transgenic rice plants were then generated through stable transformation via *Agrobacterium*-mediated cocultivation.

### Morphological Analysis

To measure the length and width of root hairs, we photographed root sections at 1 cm from apex of seminal roots at 5 DAG using a BX61 microscope (Olympus, Tokyo, Japan). NIH ImageJ (National Institute of Mental Health, Bethesda, Maryland, USA) was then used to quantitatively measure root hair length and width (Tajima and Kato 2013).

### Localization of PRX102

GFP signals were detected in the roots of transgenic plants expressing PRX102–GFP under control of the native promoter using a laser-scanning confocal microscope (Nanoscope Systems, Daejeon, Korea). For plasmolysis, roots were immersed in 500 mM mannitol.

Next, the full-length PRX102 gene was amplified and fused with pH7RWG2. *Agrobacteria* harboring the RFP–PRX102 plasmid were then infiltrated with *Agrobacterium* harboring GFP–HDEL (an ER marker), or mannosidase I–GFP (a Golgi marker), respectively, to leaves of *Nicotiana benthamiana*. Fluorescent signals were then detected 72 h after infiltration under a laser-scanning confocal microscope (Nanoscope Systems, Daejeon, Korea).

### H_2_O_2_ Staining

Peroxy Orange 1 staining was used to visualize H_2_O_2._ To do so, Peroxy Orange 1 was first dissolved in DMSO to produce a 500 μM stock. Roots were then immersed in 20 μM Peroxy Orange 1 for 15 min in the dark after which they were washed in distilled water three times.

### RNA Sequencing Analysis

To identify genes that were downregulated in the *prx102* mutant relative to the control, we performed an RNA-seq analysis. To do so we first isolated root hairs from seminal roots (Moon et al. 2018). Three biological replicates were prepared from both WT and *prx102* roots, respectively. Total RNA was then extracted with TRIzol and purified using a RNeasy Plant mini kit. RNA-seq was then performed by Macrogen Inc. on an Illumina platform. Trimmomatic version 0.39 was used to filter out adaptor sequences and low-quality bases (Bolger et al 2014). Cleaned reads were then mapped to the MSU7 rice reference genome (RGAP, http://rice.plantbiology.msu.edu/) using the HiSat2 version 2.2.1 aligner (Kim et al 2019), after which alignments were sorted using SAMTools version 1.10 (Li et al. 2009). The number of reads mapped to each gene was counted using featureCounts version 2.0.0 (Liao et al. 2014). Raw read counts were then normalized using the DESeq2 version 1.38.3 package implemented in R (Love et al. 2014). Differentially expressed genes were then identified if they met the following criteria: FPKM of WT root hair ≥ 4, log2 fold change ≤  − 1.2; and *p*-value ≤ 0.05 (Additional file 1: Table S2).

## Conclusion

We have identified a root hair-preferential endoplasmic reticulum peroxidase, *PRX102*. During root hair growth, *PRX102* localizes polarly within the tip regions of root hairs. Root hairs in *prx102* mutants are shorter and thicker compared to those in the wild type, primarily due to restricted dense cytoplasm rather than disruptions in ROS homeostasis. RNA-seq analysis of *prx102* root hairs revealed that 87 genes, including glutathione S-transferase, were downregulated. Our results suggest a novel role for peroxidase as a regulator of dense cytoplasmic streaming toward the tip.

### Supplementary Information

**Additional file 1**. **Table S1**. List of genes selected to generate mutant lines using the CRISPR-Cas9 system. **Table S2**. Selected genes found to be downregulated in* prx102* root hairs relative to WT root hairs. **Table S3**. Primer sequences used in this study.

**Additional file 2**. **Fig S1 **ROS staining of roots from the WT (A and B), prx102-1 (C and D), and prx102-4 (E and F). **Fig S2 **ER staining in roots from transgenic plants harboring the *PRX102 *promoter::PRX102–GFP construct.

**Additional file 3**. **Video S1**: Cytoplasmic streaming of root hairs in WT after initiation stage. **Video S2**: Cytoplasmic streaming of root hairs in WT at active growing stage.** Video S3**: Cytoplasmic streaming of root hairs in WT at termination of growth. **Video S4**: Cytoplasmic streaming of PRX102–GFP in WT background. **Video S5**: Cytoplasmic streaming of root hairs in *prx102-1* after initiation stage. **Video S6**: Cytoplasmic streaming of root hairs in *prx102-1* at active growing stage. **Video S7**: Cytoplasmic streaming of root hairs in *prx102-4* after initiation stage. **Video S8**: Cytoplasmic streaming of root hairs in *prx102-4* at active growing stage.

## Data Availability

All supplemental tables and figures are prepared in Additional files 1 and 2.
