# Supplementary material for: PRX102 Participates in Root Hairs Tip Growth of Rice
Source: Rice (N Y). 2023 Nov 16;16:51. doi: 10.1186/s12284-023-00668-7 (PMC10654324; doi:10.1186/s12284-023-00668-7)
Supplement: Supplementary file 1 — Additional file 1. Table S1. List of genes selected to generate mutant lines using the CRISPR-Cas9 system. Table S2. Selected genes found to be downregulated in prx102 root hairs relative to WT root hairs. Table S3. Primer sequences used in this study. [file 12284_2023_668_MOESM1_ESM.docx]

**Table S1**. List of genes selected to generate mutant lines using the CRISPR-Cas9 system.

| Locus number | Predicted gene | Target site |
| --- | --- | --- |
| LOC_Os06g37410 | helix-loop-helix DNA-binding protein | ccgggttgggcgtacaagga |
| LOC_Os03g12760 | helix-loop-helix DNA-binding protein | gcattgccaagcttggcgtt |
| LOC_Os07g31610 | peroxidase | GCGGACGGCCAGAAACAACA |
| LOC_Os01g10870 | calcium/calmodulin dependent protein kinases | ggaggtggcgatcaaggtga |

**Table S2**. Selected down-regulated genes *in prx102* root hairs compared to WT.

| Gene_ID | WT 1 | WT 2 | WT 3 | *prx102*-1 | *prx102*-2 | *prx102*-3 | fold |
| --- | --- | --- | --- | --- | --- | --- | --- |
| LOC_Os01g03680 | 3.46 | 4.08 | 2.69 | 0.24 | 0.52 | 0.03 | 1.80 |
| LOC_Os01g04620 | 18.06 | 53.45 | 48.85 | 4.52 | 5.25 | 5.78 | 2.60 |
| LOC_Os01g05610 | 25.93 | 21.43 | 31.85 | 9.14 | 10.16 | 9.28 | 1.36 |
| LOC_Os01g06010 | 29.59 | 18.51 | 18.88 | 8.23 | 9.47 | 8.86 | 1.21 |
| LOC_Os01g10210 | 7.59 | 12.30 | 11.70 | 1.63 | 2.02 | 2.28 | 1.93 |
| LOC_Os01g11230 | 21.90 | 13.15 | 11.23 | 2.90 | 5.59 | 3.63 | 1.69 |
| LOC_Os01g12340 | 1.78 | 5.12 | 4.72 | 0.58 | 0.52 | 1.26 | 1.39 |
| LOC_Os01g17330 | 26.40 | 38.69 | 31.57 | 10.31 | 12.79 | 6.86 | 1.62 |
| LOC_Os01g32670 | 256.05 | 308.29 | 242.50 | 24.98 | 38.21 | 27.41 | 3.13 |
| LOC_Os01g45110 | 32.84 | 10.14 | 16.16 | 2.00 | 2.91 | 3.43 | 2.32 |
| LOC_Os01g54670 | 10.85 | 11.55 | 8.86 | 3.76 | 5.23 | 2.45 | 1.28 |
| LOC_Os01g55510 | 58.28 | 82.10 | 61.14 | 19.86 | 28.05 | 21.54 | 1.50 |
| LOC_Os01g58970 | 1.51 | 4.52 | 4.28 | 0.72 | 1.01 | 0.29 | 1.35 |
| LOC_Os01g72130 | 207.05 | 104.80 | 109.41 | 17.61 | 29.43 | 58.04 | 2.06 |
| LOC_Os02g28334 | 24.02 | 13.30 | 16.31 | 2.39 | 3.36 | 1.98 | 2.38 |
| LOC_Os02g40500 | 100.04 | 71.39 | 76.59 | 30.00 | 39.79 | 26.18 | 1.35 |
| LOC_Os03g03410 | 14.48 | 6.10 | 9.02 | 2.00 | 1.93 | 1.81 | 1.83 |
| LOC_Os03g17100 | 6.95 | 2.31 | 2.49 | 0.34 | 0.35 | 1.08 | 1.54 |
| LOC_Os03g17470 | 59.19 | 75.91 | 58.32 | 11.62 | 16.06 | 26.55 | 1.84 |
| LOC_Os03g25300 | 5.49 | 8.70 | 14.79 | 2.54 | 2.97 | 0.97 | 1.72 |
| LOC_Os03g29410 | 7.31 | 3.94 | 3.00 | 0.58 | 0.68 | 2.36 | 1.40 |
| LOC_Os03g56060 | 11.03 | 16.36 | 14.50 | 4.62 | 5.34 | 5.62 | 1.26 |
| LOC_Os04g12560 | 7.78 | 7.06 | 5.15 | 1.13 | 1.07 | 1.27 | 1.81 |
| LOC_Os04g27060 | 19.27 | 11.04 | 9.13 | 1.49 | 2.81 | 2.52 | 2.07 |
| LOC_Os04g37490 | 14.53 | 6.10 | 8.65 | 0.71 | 1.66 | 3.90 | 1.86 |
| LOC_Os04g39320 | 23.78 | 7.65 | 13.76 | 2.58 | 2.77 | 4.27 | 1.83 |
| LOC_Os04g40630 | 33.70 | 44.82 | 39.61 | 14.39 | 13.63 | 8.56 | 1.64 |
| LOC_Os04g41570 | 7.49 | 3.05 | 3.11 | 0.67 | 0.95 | 1.97 | 1.29 |
| LOC_Os04g53606 | 3.49 | 6.64 | 3.70 | 0.43 | 0.40 | 0.85 | 1.82 |
| LOC_Os04g56420 | 40.36 | 17.18 | 15.28 | 3.77 | 8.13 | 6.72 | 1.73 |
| LOC_Os04g56430 | 347.66 | 2121.20 | 1980.62 | 85.73 | 55.94 | 167.49 | 3.59 |
| LOC_Os04g58280 | 15.37 | 46.83 | 37.75 | 5.33 | 8.65 | 8.16 | 1.92 |
| LOC_Os05g15150 | 4.34 | 4.74 | 4.06 | 0.95 | 0.82 | 1.13 | 1.45 |
| LOC_Os05g30720 | 2.29 | 4.61 | 4.05 | 0.00 | 0.00 | 0.00 | 2.18 |
| LOC_Os05g33430 | 3.16 | 10.67 | 10.32 | 0.64 | 1.71 | 1.37 | 1.90 |
| LOC_Os05g33570 | 5.69 | 7.96 | 7.16 | 0.61 | 1.13 | 2.03 | 1.85 |
| LOC_Os05g34710 | 4.48 | 9.45 | 8.62 | 2.73 | 3.14 | 1.25 | 1.33 |
| LOC_Os05g36270 | 12.14 | 14.48 | 12.74 | 0.93 | 1.94 | 2.47 | 2.38 |
| LOC_Os05g39770 | 8.51 | 6.41 | 6.32 | 1.19 | 2.06 | 3.25 | 1.39 |
| LOC_Os05g45100 | 29.91 | 31.86 | 30.03 | 7.00 | 6.83 | 11.85 | 1.76 |
| LOC_Os05g49860 | 11.63 | 6.22 | 5.87 | 1.64 | 2.45 | 1.30 | 1.63 |
| LOC_Os06g13190 | 166.76 | 241.76 | 226.86 | 83.31 | 85.08 | 94.15 | 1.25 |
| LOC_Os06g16050 | 4.58 | 8.29 | 10.07 | 0.00 | 0.00 | 0.00 | 3.05 |
| LOC_Os06g35520 | 27.30 | 58.37 | 45.23 | 14.97 | 24.43 | 11.82 | 1.30 |
| LOC_Os06g35700 | 41.13 | 87.58 | 95.86 | 29.37 | 29.90 | 14.28 | 1.55 |
| LOC_Os06g36560 | 64.68 | 167.03 | 145.60 | 24.37 | 34.38 | 18.77 | 2.17 |
| LOC_Os06g50950 | 5.95 | 6.09 | 5.42 | 1.70 | 1.83 | 2.35 | 1.21 |
| LOC_Os07g23570 | 27.24 | 7.19 | 20.68 | 1.70 | 2.48 | 5.72 | 2.10 |
| LOC_Os07g26110 | 131.17 | 314.44 | 337.51 | 53.12 | 52.84 | 47.43 | 2.21 |
| LOC_Os07g43260 | 6.66 | 8.68 | 6.66 | 0.33 | 0.22 | 0.86 | 2.52 |
| LOC_Os07g44770 | 12.66 | 18.19 | 30.99 | 7.81 | 7.32 | 4.33 | 1.47 |
| LOC_Os08g09010 | 624.62 | 541.94 | 728.13 | 61.75 | 92.23 | 114.46 | 2.84 |
| LOC_Os08g09040 | 8.40 | 37.46 | 46.49 | 0.85 | 0.59 | 6.27 | 3.22 |
| LOC_Os08g23780 | 2.79 | 3.79 | 3.92 | 0.53 | 0.75 | 0.99 | 1.36 |
| LOC_Os08g26870 | 8.42 | 17.77 | 13.34 | 3.86 | 3.94 | 5.99 | 1.31 |
| LOC_Os08g37670 | 144.68 | 169.59 | 144.94 | 38.93 | 61.54 | 68.54 | 1.46 |
| LOC_Os09g15510 | 9.12 | 19.15 | 16.58 | 4.24 | 5.99 | 2.57 | 1.59 |
| LOC_Os09g17530 | 4.16 | 6.53 | 4.44 | 0.32 | 0.55 | 0.87 | 1.93 |
| LOC_Os09g31019 | 37.27 | 60.45 | 51.80 | 9.25 | 15.80 | 3.74 | 2.42 |
| LOC_Os09g31031 | 21.26 | 27.78 | 22.12 | 3.61 | 5.28 | 4.51 | 2.18 |
| LOC_Os10g19200 | 7.60 | 7.47 | 8.66 | 2.91 | 3.60 | 1.83 | 1.26 |
| LOC_Os10g34020 | 11.27 | 3.85 | 6.93 | 0.93 | 1.73 | 1.88 | 1.65 |
| LOC_Os10g34930 | 14.24 | 6.72 | 7.04 | 0.63 | 0.93 | 3.35 | 2.04 |
| LOC_Os10g40440 | 24.62 | 14.67 | 15.92 | 4.28 | 10.69 | 3.47 | 1.54 |
| LOC_Os11g02240 | 22.88 | 15.60 | 13.55 | 2.83 | 4.99 | 2.43 | 2.06 |
| LOC_Os11g03970 | 4.34 | 18.10 | 16.81 | 0.15 | 0.18 | 0.18 | 3.38 |
| LOC_Os11g04920 | 2.20 | 3.69 | 3.74 | 0.00 | 0.00 | 0.00 | 2.05 |
| LOC_Os11g14544 | 18.67 | 25.31 | 22.28 | 6.59 | 8.07 | 10.31 | 1.32 |
| LOC_Os11g17970 | 4.55 | 11.14 | 9.94 | 0.59 | 0.78 | 3.86 | 1.92 |
| LOC_Os11g29400 | 189.54 | 163.88 | 186.93 | 77.73 | 85.13 | 66.28 | 1.23 |
| LOC_Os11g32810 | 3.02 | 4.32 | 3.82 | 0.00 | 0.00 | 0.00 | 2.23 |
| LOC_Os11g34270 | 17.51 | 22.81 | 14.09 | 4.26 | 4.76 | 4.89 | 1.74 |
| LOC_Os11g41640 | 19.13 | 15.94 | 21.35 | 5.38 | 7.60 | 4.47 | 1.56 |
| LOC_Os11g47400 | 4.75 | 12.35 | 12.25 | 0.00 | 0.00 | 0.00 | 3.33 |
| LOC_Os12g03230 | 10.51 | 18.85 | 23.04 | 5.17 | 7.42 | 5.07 | 1.37 |
| LOC_Os12g03240 | 4.14 | 7.95 | 10.57 | 1.82 | 2.45 | 1.73 | 1.44 |
| LOC_Os12g05840 | 31.67 | 16.06 | 32.78 | 1.98 | 4.24 | 6.63 | 2.43 |
| LOC_Os12g05860 | 23.68 | 8.03 | 18.67 | 1.46 | 2.58 | 3.37 | 2.28 |
| LOC_Os12g05870 | 18.93 | 5.96 | 13.50 | 0.77 | 0.69 | 3.86 | 2.37 |
| LOC_Os12g13674 | 13.62 | 58.84 | 55.79 | 0.00 | 0.00 | 0.00 | 5.20 |
| LOC_Os12g15222 | 4.27 | 6.50 | 5.85 | 0.00 | 0.01 | 0.00 | 2.69 |
| LOC_Os12g15505 | 5.17 | 9.26 | 10.41 | 0.00 | 0.00 | 0.00 | 3.17 |
| LOC_Os12g16200 | 7.69 | 8.43 | 14.10 | 1.62 | 1.59 | 0.64 | 2.27 |
| LOC_Os12g17950 | 9.33 | 5.60 | 11.99 | 0.63 | 0.82 | 1.52 | 2.30 |
| LOC_Os12g21590 | 3.29 | 4.68 | 5.15 | 0.64 | 0.83 | 0.16 | 1.81 |
| LOC_Os12g22810 | 5.24 | 7.99 | 7.07 | 0.08 | 0.04 | 0.00 | 2.89 |
| LOC_Os12g25630 | 16.25 | 29.04 | 23.75 | 0.74 | 1.17 | 2.71 | 3.28 |

| **Table S3**. Primer sequences used in this study. | |
| --- | --- |
| Primer name | Primer sequence |
| HLH030 Cas-F | GGCACCGGGTTGGGCGTACAAGGA |
| HLH030 Cas-R | AAACTCCTTGTACGCCCAACCCGG |
| HLH019 Cas-F | GGCAGCATTGCCAAGCTTGGCGTT |
| HLH019 Cas-R | AAACAACGCCAAGCTTGGCAATGC |
| CIPK13 Cas-F | GGCAGGAGGTGGCGATCAAGGTGA |
| CIPK13 Cas-R | AAACTCACCTTGATCGCCACCTCC |
| PRX102 cas 1F | GGCAGCGGACGGCCAGAAACAACA |
| PRX102 cas 1R | AAACTGTTGTTTCTGGCCGTCCGC |
| PRX102 cas 2 F | GGCACTACTACCACCAGTCGTGCC |
| PRX102 cas 2 R | AAACGGCACGACTGGTGGTAGTAG |
| PRX102 full F | GCTTGTTAACACTAGTAAAACAAAAAATGACGAGGACGCC |
| PRX102 full R | CCTTGCTCACACTAGTGTAGGAGTTGCTGTTGATGGCG |
| PRX102 promoter F | CGCCCGGATCAAGCTGCTTCGAATTGACAAGTCCATTG |
| PRX102 promoterR | TAGTGTTAACAAGCTCTTGATCGATCGAAAGGGAAAAC |
| PRX102 GFP F | GCTCACTAGTTCTAGAacgaggacgccggcgtctct |
| PRX102 GFP R | AATTATCTAGTCTAGAttgacattactcgcataagc |
| GST1F | CAACCGATCCGTACGATCGA |
| GST1R | CCTTGAGCTGCCCTTCAAGA |
| GST2F | CTCTGAGAAGGAAGTGCTCC |
| GST2R | CTTGTAGTTCCTGGCAATCC |
| GST3F | GACAGTACATCGACGACATG |
| GST3R | GCTGCACTCGACGAATGCCT |
| PRX102 amplify F | AATGCACAGCTCACGGCAGC |
| PRX102 amplify R | TTGACATTACTCGCATAAGC |
| HLH030 amplify F | CGTGCGCAGCCATCTGTTCT |
| HLH030 amplify R | CCCGCTGTCGCATCCCATGA |
| HLH019 amplify F | GCTCTGAAATCTTCGAGCAA |
| HLH019 amplify R | CATGATCGGGGCACATGTAC |
| CIPK13 amplify F | CCTAGCACACTCTCCATCAT |
| CIPK13 amplify R | CCGCGGCGCGACAGCACCTC |
| PRX102 RT F | CTACTACCACCAGTCGTGCC |
| PRX102 RT R | TGGACTCGATGAGCTCGAAC |
