# Supplementary material for: PRX102 Participates in Root Hairs Tip Growth of Rice
Source: Rice (N Y). 2023 Nov 16;16:51. doi: 10.1186/s12284-023-00668-7 (PMC10654324; doi:10.1186/s12284-023-00668-7)
Supplement: Supplementary file 2 — Additional file 2. Fig S1 ROS staining of roots from the WT (A and B), prx102-1 (C and D), and prx102-4 (E and F). Fig S2 ER staining in roots from transgenic plants harboring the PRX102 promoter::PRX102–GFP construct. [file 12284_2023_668_MOESM2_ESM.docx]

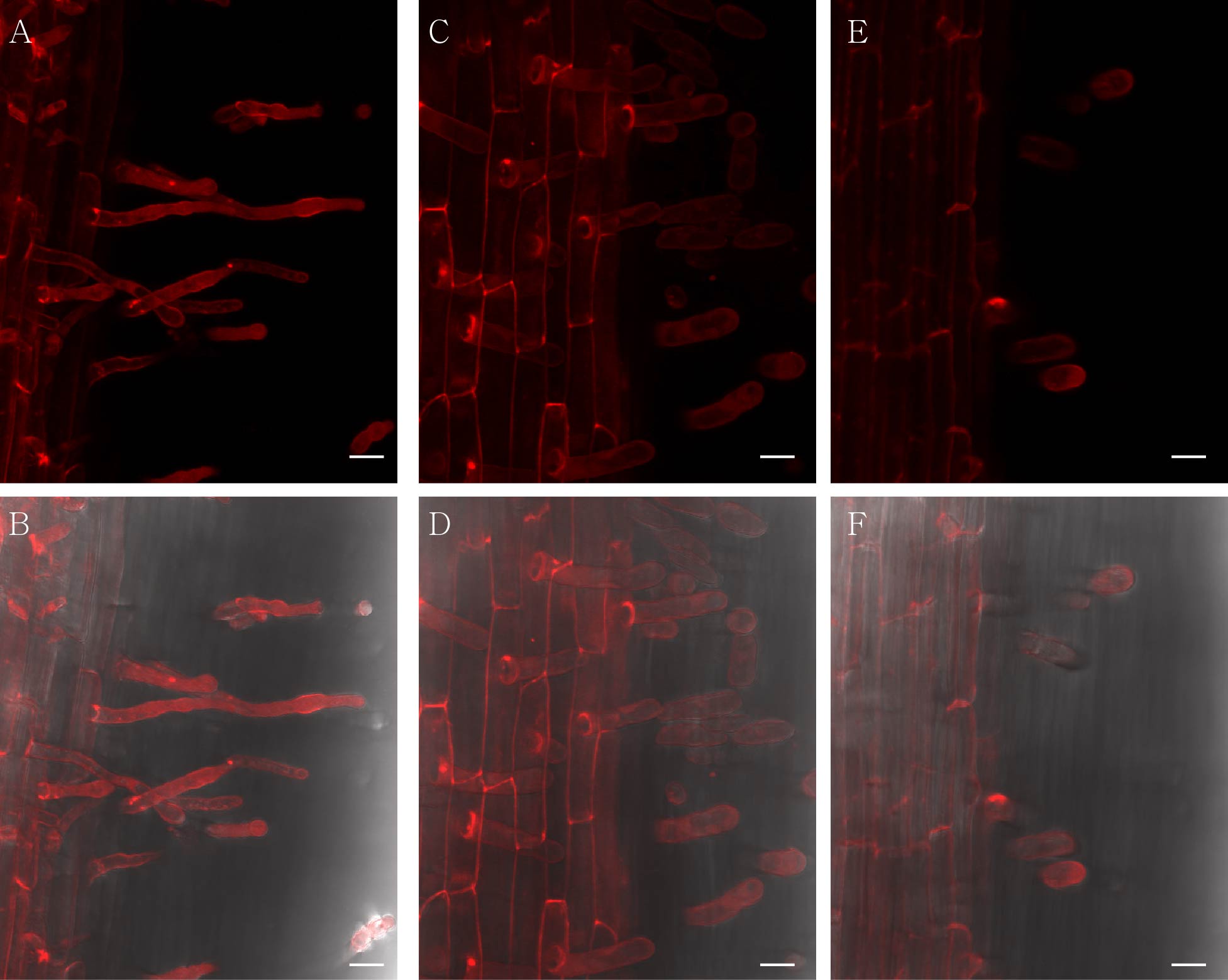


**Fig S1** ROS staining of roots from wild type control (A and B), *prx102-1* (C and D), and *prx102-4* (E and F).


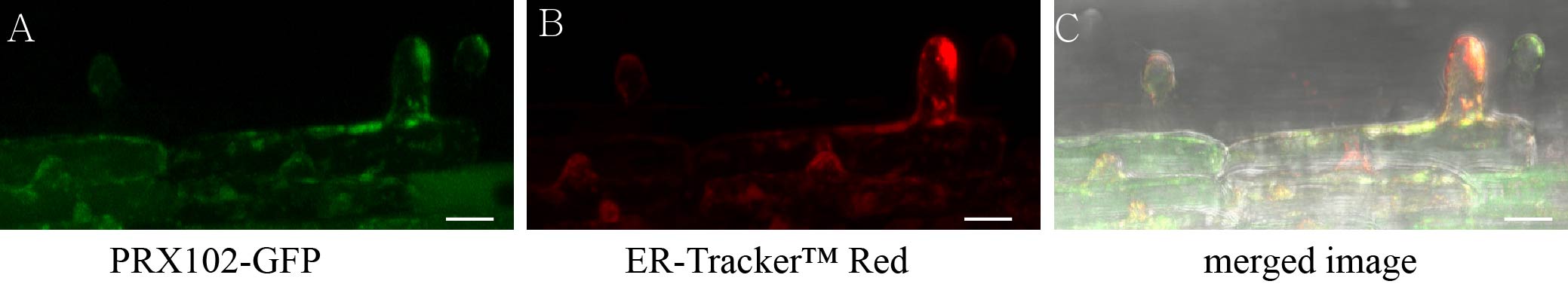


**Fig S2** ER staining in roots from transgenic plants harboring the *PRX102* promoter::PRX102–GFP.
